# *Vm‐*milR37 contributes to pathogenicity by regulating glutathione peroxidase gene *VmGP* in *Valsa mali*

**DOI:** 10.1111/mpp.13023

**Published:** 2020-12-05

**Authors:** Hao Feng, Ming Xu, Yuqi Gao, Jiahao Liang, Feiran Guo, Yan Guo, Lili Huang

**Affiliations:** ^1^ State Key Laboratory of Crop Stress Biology for Arid Areas and College of Plant Protection Northwest A&F University Yangling China; ^2^ State Key Laboratory of Crop Stress Biology for Arid Areas and College of Life Sciences Northwest A&F University Yangling China

**Keywords:** apple tree, microRNA‐like RNA, oxidative stress, posttranscriptional regulation virulence Valsa canker

## Abstract

MicroRNAs play important roles in various biological processes by regulating their corresponding target genes. However, the function and regulatory mechanism of fungal microRNA‐like RNAs (milRNAs) are still largely unknown. In this study, a milRNA (*Vm‐*milR37) was isolated and identified from *Valsa mali*, which causes the most serious disease on the trunk of apple trees in China. Based on the results of deep sequencing and quantitative reverse transcription PCR, *Vm‐*milR37 was found to be expressed in the mycelium, while it was not expressed during the *V. mali* infection process. Overexpression of *Vm‐*milR37 did not affect vegetative growth, but significantly decreased pathogenicity. Based on degradome sequencing, the target of *Vm‐*milR37 was identified as *VmGP*, a glutathione peroxidase. The expression of *Vm‐*milR37 and *VmGP* showed a divergent trend in *V. mali–*apple interaction samples and *Vm‐*milR37 overexpression transformants. The expression of *VmGP* could be suppressed significantly by *Vm‐*milR37 when coexpressed in tobacco leaves. Deletion of *VmGP* showed significantly reduced pathogenicity compared with the wild type. *VmGP* deletion mutants showed more sensitivity to hydrogen peroxide. Apple leaves inoculated with *Vm*‐milR37 overexpression transformants and *VmGP* deletion mutant displayed increased accumulation of reactive oxygen species compared with the wild type. Thus, *Vm*‐milR37 plays a critical role in pathogenicity by regulating *VmGP*, which contributes to the oxidative stress response during *V. mali* infection. These results provide important evidence to define the roles of milRNAs and their corresponding target genes in pathogenicity.

## Graphical abstract

This study demonstrates that a fungal milRNA *Vm*‐milR37 contributes to pathogenicity by adaptively regulating the glutathione peroxidase gene (*VmGP*), which contributes to the oxidative stress response in *Valsa mali*.

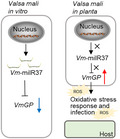

## 1 INTRODUCTION

The RNA interference (RNAi) pathway, a conserved regulatory mechanism in eukaryotes first described in *Caenorbabditis elegans*, is a phenomenon triggered by small RNAs (sRNAs) that are generated from double‐stranded RNA (dsRNA) by Dicer or Dicer‐like (DCL) proteins (Fire et al., 1998). There are three main classes of sRNAs: small interfering RNAs (siRNAs), microRNAs (miRNAs), and PIWI‐interacting RNAs (piRNAs) (Carthew & Sontheimer, 2009; Thomson & Lin, 2009). The sRNAs are loaded into Argonaute (AGO) proteins, which are the core component of the RNA‐induced silencing complex (RISC). A guide RNA directs the RISC to complementary message RNAs (mRNAs), resulting in mRNA cleavage or repression of translation (Chang et al., 2012; Holoch & Moazed, 2015). Various studies have shown that the RNAi pathway plays important roles in growth, development, reproduction, and response to biotic or abiotic stresses in eukaryotes (Ghildiyal & Zamore, 2009; Katiyar‐Agarwal & Jin, 2010).

The first RNAi description in fungi was reported in *Neurospora* (Romano & Macino, 1992). Since then, the identification and characterization of RNAi components have deepened our understanding of fungal RNAi (Nakayashiki et al., 2006). There is evidence that fungal RNAi plays important roles in maintenance of growth, antiviral defence, sexual development, and pathogenicity (Jin et al., 2019; Raman et al., 2017; Son et al., 2017; Sun et al., 2009; Torres‐Martínez & Ruiz‐Vázquez, 2017; Weiberg et al., 2013). However, the detailed mechanisms of regulation by sRNAs in fungi are still largely not understood.

miRNAs are 21‐nucleotide endogenous RNAs generated from single‐stranded RNA with a hairpin structure. The various functions and corresponding regulatory mechanism of miRNAs in plants and animals have been reported (Bartel, 2004; Grimson et al., 2008; Llave et al., 2002). However, it was believed that miRNAs were absent in fungi. In 2010 a class of sRNAs that have a similar generation pathway and regulatory mechanism to miRNAs in plants and animals was identified in *Neurospora* and designated as microRNA‐like RNAs (milRNA) (Lee et al., 2010). Further milRNAs were isolated and functionally analysed in fungi by the application of deep‐sequencing technology. In *Metarhizium anisopliae* and *Trichoderma reesei*, milRNAs were predicted to be related to mycelial growth and sporulation (Kang et al., 2013; Zhou et al., 2012b). The milRNAs of *Penicillium marneffei* regulate the growth process of mycelial and yeast phases (Lau et al., 2013). milRNAs are speculated to be important regulators for toxin biosynthesis in *Aspergillus flavus* (Bai et al., 2015).

In recent years, the function of milRNAs isolated from plant‐pathogenic fungi have been analysed. In *Sclerotinia sclerotiorum*, 44 candidate milRNAs were identified and predicted to be associated with sclerotial development (Zhou et al., 2012a). milRNAs of *Fusarium oxysporum* f. sp. *niveum* play important roles in the biosynthesis of fungal toxins (Jiang et al., 2017). In *Rhizoctonia solani*, several milRNAs affect pathogenicity by regulating many important pathogenic factors (Lin et al., 2016). In addition, studies of milRNAs and their targets in *Curvularia lunata*, *F. oxysporum*, *Zymoseptoria tritici*, and *Puccinia striiformis* f. sp. *tritici* suggested that milRNAs were also associated with pathogenicity and development (Chen et al., 2014; Liu et al., 2016; Mueth et al., 2015; Yang, 2015). However, the function of most milRNAs was only based on the target prediction; the detailed regulatory mechanism of milRNAs was not elucidated. Recent studies on *Verticillium dahliae* demonstrated that VdmilR1 can suppress target gene expression by epigenetic repression to regulate pathogenicity (Jin et al., 2019). *Pst*‐milRNA1 was found to contribute to pathogenicity by suppressing the expression of the host wheat pathogenesis‐related 2 gene (Wang et al., 2017a). Thus, fungal milRNAs may have multiple functions by regulating different targets, and it is of great interest to identify the regulatory mechanism of different milRNAs.

*Valsa mali* is an important phytopathogenic fungus, causing the most serious trunk disease of apple trees (Wang et al., 2014). Revealing the pathogenic mechanism of *V. mali* will lay a foundation for the development of sustainable disease control strategies. Several pathogenicity factors have been characterized based on genome, transcriptome, and functional genomics (Ke et al., 2014; Wu et al., 2018; Xu et al., 2018; Yin et al., 2015; Zhang et al., 2019). The RNAi pathway components of *V. mali*, such as Dicer‐like and AGO, were identified as associated with pathogenicity, which indicated posttranscriptional regulation may also be an important pathway (Feng et al., 2017a,b). Multiple omics analyses revealed that *Vm*‐milRNAs can regulate pathogenicity factors to promote *V. mali* infection (Xu et al., 2020). However, the detailed regulatory mechanism of *Vm*‐milRNAs is still largely not understood.

In this study, *Vm‐*milR37 was specifically expressed in mycelia, but poorly expressed during infection. Functional analysis of *Vm‐*milR37 showed that it was negatively involved in pathogenicity. Its target was confirmed to be a glutathione peroxidase gene, *VmGP*, based on degradome sequencing and cotransformation results. *VmGP* was confirmed to contribute positively to pathogenicity. This study indicates that *Vm‐*milR37 contributes to pathogenicity by enhancing the expression of *VmGP* during *V. mali* infection. The results help to uncover the posttranscriptional regulatory mechanism directed by milRNAs of *V. mali*.

## 2 RESULTS

### 2.1 *Vm*‐milR37 shows expression in mycelia but no expression during *V. mali* infection

**FIGURE 1:**
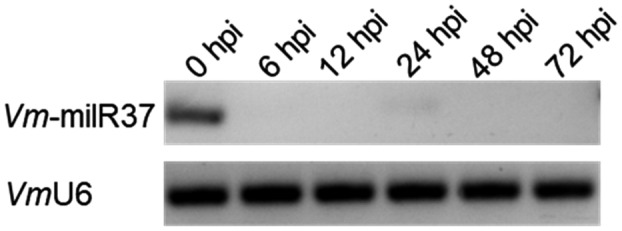
Expression patterns of *Vm*‐milR37 detected by stem‐loop reverse transcription PCR. Small nuclear RNA U6 (*Vm*U6) of *Valsa mali* was used as the internal control. *Vm*‐milR37 was expressed in vitro in mycelia and was very weakly expressed during *V. mali* infection. Similar results were observed in three biological repeats

In our previous study, *Vm*‐milR37 was isolated from a cDNA library of the mycelium of *V. mali*, which was generated from a precursor with a typical hairpin structure (Figure S1). Almost no reads of *Vm*‐milR37 were detected in the cDNA library of the *V. mali–*apple interaction. To determine whether *Vm*‐milR37 is involved in the pathogenicity of *V*. *mali*, the expression trend of *Vm*‐milR37 during *V. mali* infection was analysed by stem‐loop reverse transcription PCR. Consistent with the sequencing results, *Vm*‐milR37 was expressed in mycelia at a high level, but showed nearly no expression during *V. mali* infection (Figure 1). The result indicates a potential role in the regulation of pathogenicity of *V. mali*.

### 2.2 Overexpression of *Vm*‐milR37 decreased the pathogenicity of *V. mali*

**FIGURE 2:**
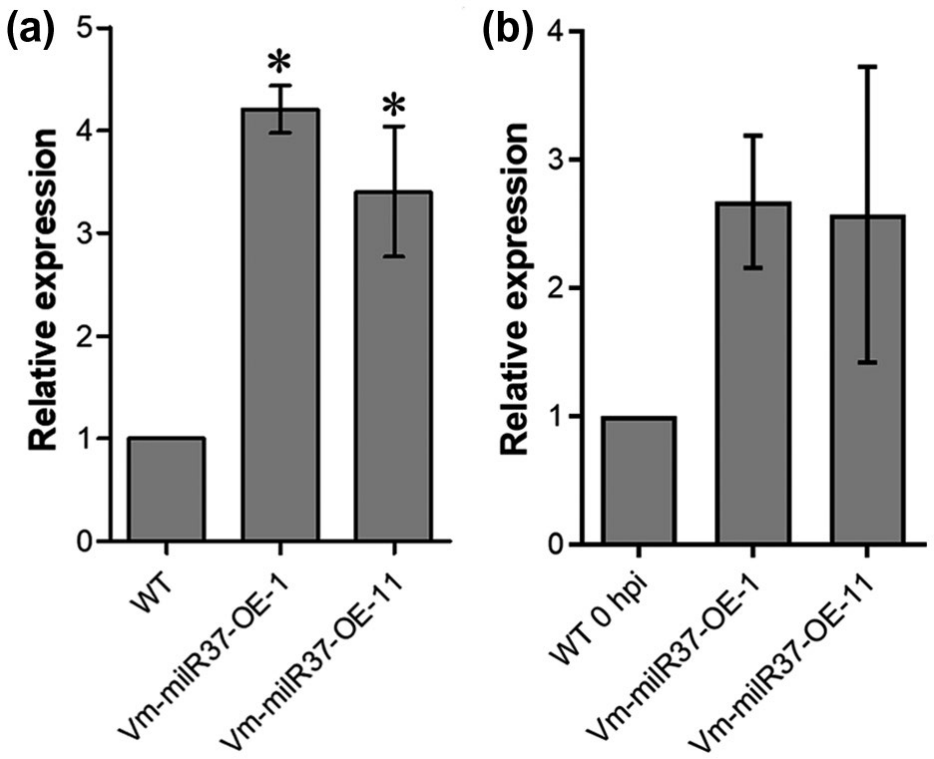
Relative expression level of *Vm*‐milR37 in *Vm*‐milR37 overexpression transformants in vitro and in planta. (a) *Vm*‐milR37 was overexpressed in *Vm*‐milR37 overexpression (OE) transformants in mycelia cultured in vitro. Total RNA of mycelia was extracted and the transcript level of *Vm*‐milR37 was detected by stem‐loop reverse transcription PCR. Relative expression of *Vm*‐milR37 was normalized to internal control *Valsa mali* small nuclear RNA U6 (*Vm*U6) and calibrated to the levels of the wild‐type strain (WT) by the 2^−ΔΔ^ *^C^* ^t^ method. (b) *Vm*‐milR37 showed enhanced transcription level in *Vm*‐milR37 overexpression transformant‐infected apple bark tissues at 24 hr postinoculation (hpi). Apple bark tissues inoculated with the WT mycelia at 0 hr postinoculation (hpi) was used as the control. In (a) and (b), mean ± *SD* was calculated from three independent biological repeats. Data were analysed using Dunnett's multiple comparison test. *p < .05

**FIGURE 3:**
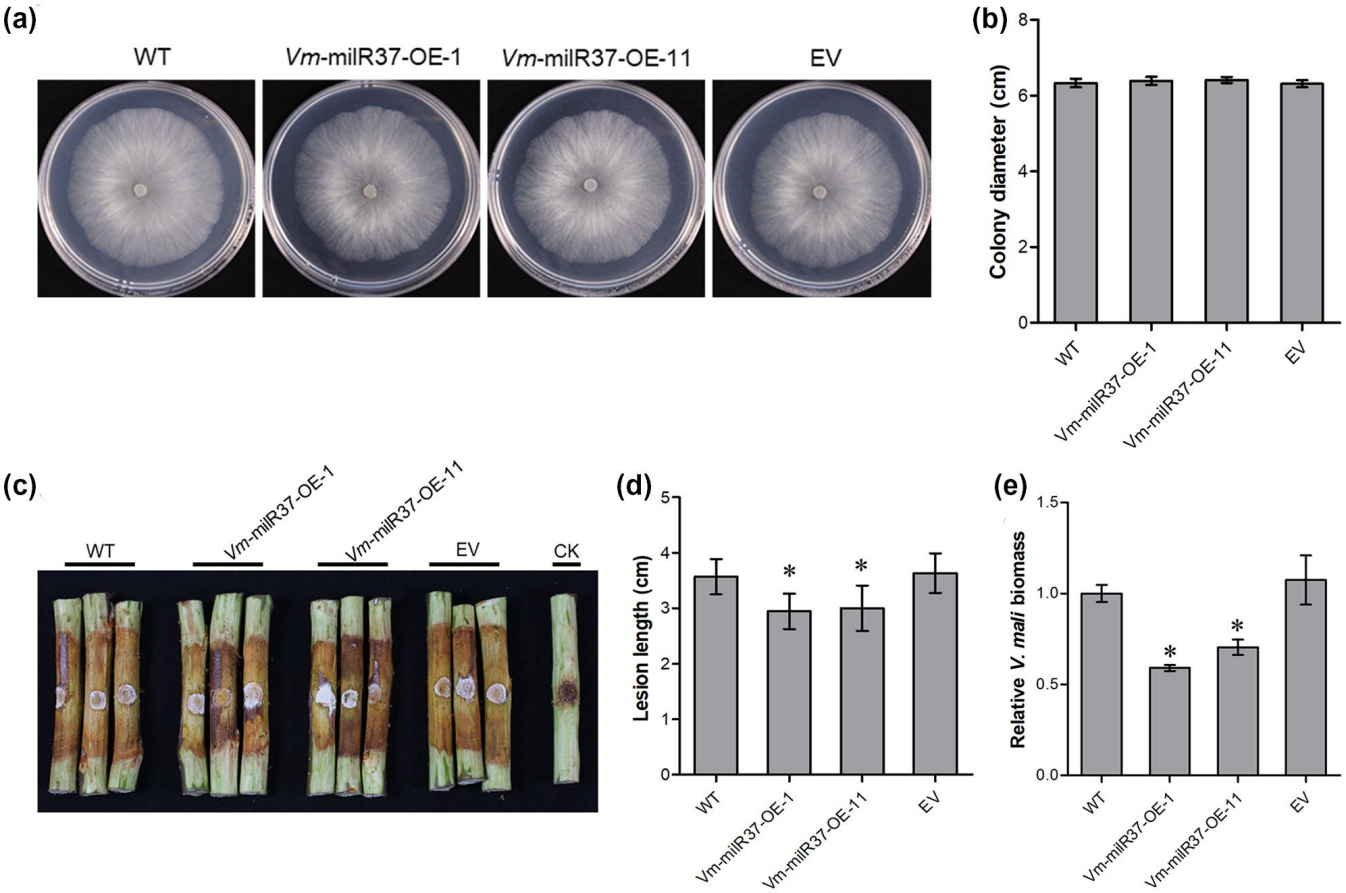
Overexpression of *Vm*‐milR37 reduces the pathogenicity of *Valsa mali*. (a) Colony morphology of wild‐type (WT), *Vm*‐milR37 overexpression (OE) transformants and empty vector transformant (EV) after 48 hr incubation. (b) Colony diameters of WT, *Vm*‐milR37 overexpression transformants, and EV after 48 hr incubation. Data represent mean ± *SD*. The experiment was repeated three times, each time with three plates. (c) and (d) Pathogenicity test of WT, *Vm*‐milR37 overexpression transformants, and EV at 4 days postinoculation. Three representative diseased twigs are shown. The pathogenicity test was independently repeated three times, each time with four replicates. CK, negative control. Data represent mean ± *SD*. (e) *V. mali* biomass was measured with quantitative PCR using *V*. *mali*‐specific *VmG6PDH* primers. Data are mean ± *SD* of three technical replicates. Similar results were obtained from three biological repeats. Significant difference was analysed using Dunnett's multiple comparison test. *p < .05

To further examine the function of *Vm*‐milR37 on the pathogenicity of *V. mali*, *Vm*‐milR37 overexpression transformants were generated (Figure S2). Two randomly selected *Vm*‐milR37 overexpression transformants (*Vm*‐milR37‐OE‐1 and *Vm*‐milR37‐OE‐11) were selected for further analysis. Compared with the wild type, the expression levels of *Vm*‐milR37 in *Vm*‐milR37‐OE‐1 and *Vm*‐milR37‐OE‐11 were enhanced 4.3‐ and 3.5‐fold, respectively (Figure 2a). *Vm*‐milR37 also showed enhanced transcription levels during infection by *Vm*‐milR37 overexpression transformants (Figure 2b). Further analysis showed that overexpression of *Vm*‐milR37 did not affect vegetative growth (Figure 3a,b). However, overexpression of *Vm*‐milR37 significantly reduced the pathogenicity of *V. mali*. Lesions caused by *Vm*‐milR37 overexpression transformants were much smaller than those caused by the wild‐type and the empty vector transformant (Figure 3c,d). The biomass of *Vm*‐milR37 overexpression transformants in twigs was significantly less than in twigs infected with the wild‐type and the empty vector transformant (Figure 3e). These results indicate that *Vm*‐milR37 might play a negative role in pathogenicity of *V. mali*.

### 2.3 Isolation and annotation of target gene of *Vm‐*milR37

In a previous study, VM1G_06866, which encodes a glutathione peroxidase, a protein of 229 amino acids with typical glutathione peroxidase conserved domains (Figure S3), was identified as a target gene of *Vm*‐milR37 (Xu et al., 2020). VM1G_06866 was designated as *VmGP*. VM1G_06866 is predicted by BLAST searches with the pathogen–host interactions database (PHI‐base) to possibly be a virulence gene (Urban et al., 2017). We speculated that *Vm*‐milR37 could be involved in pathogenicity by regulating the expression of VM1G_06866.

VmGP was confirmed to be a unique glutathione peroxidase in *V*. *mali* by searching the nonredundant protein sequence database (nr) using BlastP (https://blast.ncbi.nlm.nih.gov/Blast.cgi?PROGRAM=blastp&PAGE_TYPE=BlastSearch&LINK_LOC=blasthome) (Figure S4). We also analysed the protein sequence using SignalP v. 5.0 (http://www.cbs.dtu.dk/services/SignalP‐5.0/); no signal peptide was found in VmGP (Figure S5). We predicted the protein subcellular localization of VmGP using WoLF PSORT (https://www.genscript.com/wolf‐psort.html?src=leftbar); VmGP is likely to be located in the mitochondrion (Figure S6).

**FIGURE 4:**
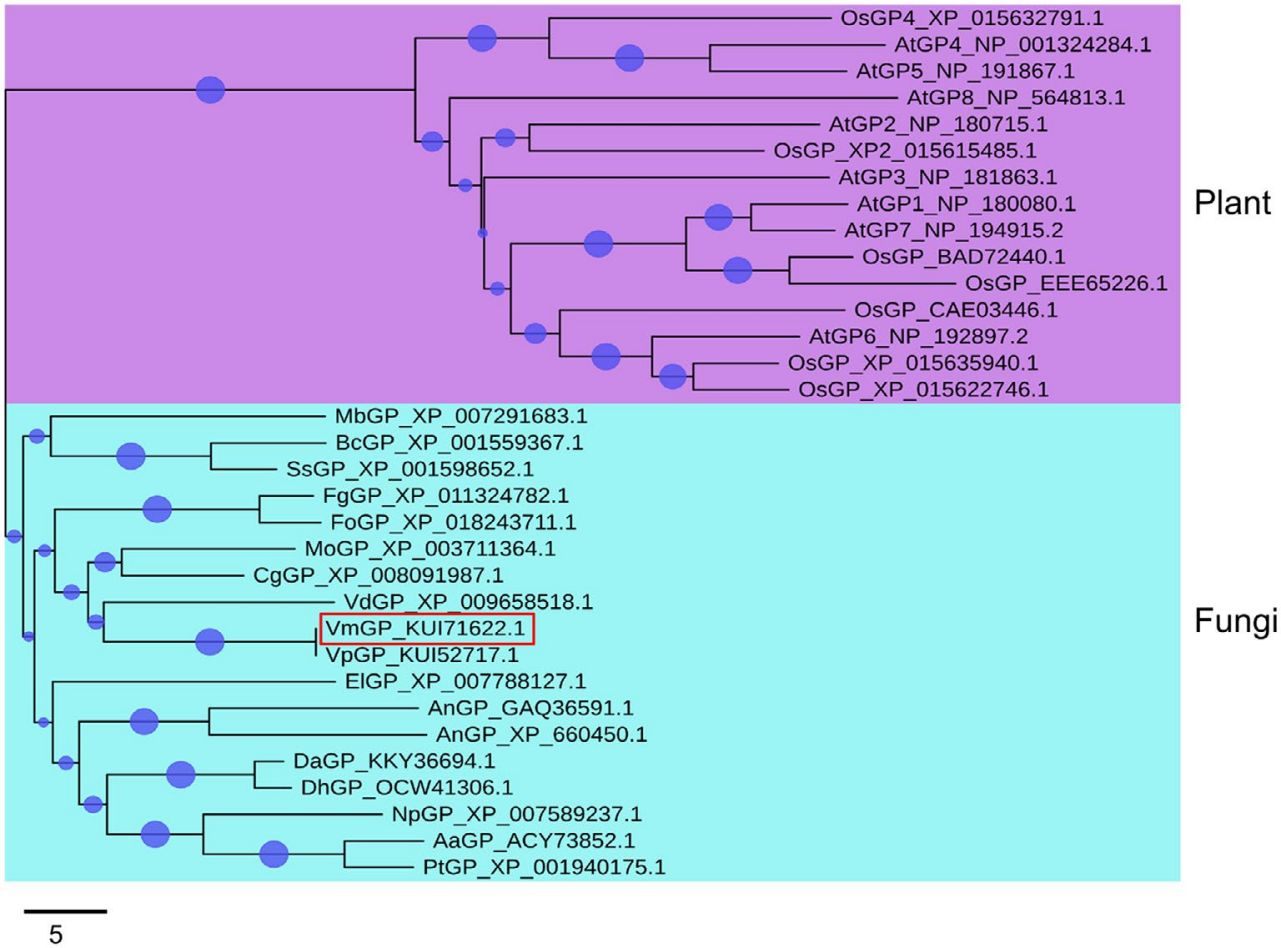
Phylogenetic analysis of VmGP. The phylogenetic tree was constructed with neighbour‐joining method using MEGA 7. Bootstrap values were set as 1,000. VmGP is highlighted in a red box. At, *Arabidopsis thaliana*; Os, *Oryza sativa*; Mb, *Marssonina brunnea*; Bc, *Botrytis cinerea*; Ss, *Sclerotinia sclerotiorum*; Fg, *Fusarium graminearum*; Fo, *Fusarium oxysporum*; Mo, *Magnaporthe oryzae*; Cg, *Colletotrichum graminicola*; Vd, *Verticillium dahliae*; Vm, *Valsa mali*; Vp, *Valsa pyri*; El, *Eutypa lata*; An, *Aspergillus niger*; Da, *Diaporthe ampelina*; Dh, *Diaporthe helianthi*; Np, *Neofusicoccum parvum*; Aa, *Alternaria alternata*; Pt, *Pyrenophora tritici‐repentis*

To study the phylogeny of glutathione peroxidase, 15 homologous plant proteins and 18 homologous fungal proteins were identified and used to establish a neighbour‐joining phylogenetic tree (Figure 4). VmGP (KUI71622) clustered with the glutathione peroxidase from fungi as expected, and it was highly homologous with the glutathione peroxidase of *Valsapyri* (KUI52717) and *V. dahliae* (XP_009658518). The glutathione peroxidase proteins from plants clustered together and the glutathione peroxidase proteins from fungi clustered together, suggesting a common evolutionary origin.

### 2.4 Validation of the target gene of *Vm*‐milR37

**FIGURE 5:**
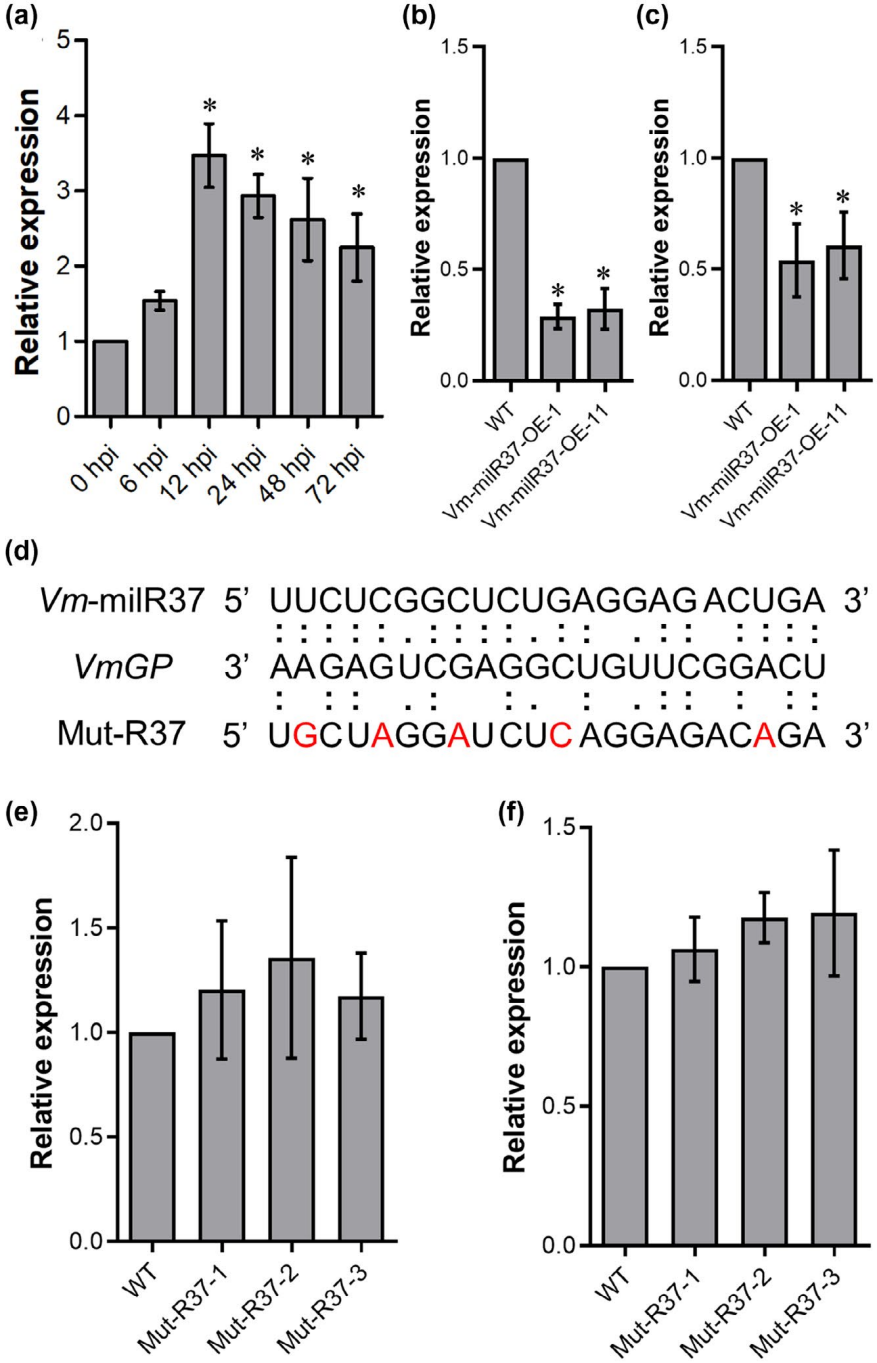
Relative expression of *VmGP* detected by quantitative reverse transcription PCR. (a) The relative expression of *VmGP* during *Valsa mali* infection. (b) Relative expression of *VmGP* in *Vm*‐milR37 overexpression transformants in mycelia in vitro. The relative expression was normalized to the reference gene *VmG6PDH* calibrated to the levels of 0 hr postinoculation (hpi)/wild type (WT) (set as 1) by the 2^−ΔΔ^ *^C^* ^t^ method. (c) The expression of *VmGP* was suppressed at 24 hr after *Vm*‐milR37 overexpression transformant inoculation. (d) Sequence alignment of *VmGP* with *Vm*‐milR37 and mutated *Vm*‐milR37 (Mut‐R37). (e) and (f) The expression of *VmGP* was not suppressed in Mut‐R37 overexpression transformants (Mut‐R37‐1, Mut‐R37‐2, and Mut‐R37‐3) in cultured mycelia in vitro (e) and in planta (24 hpi) (f). *SD* was calculated from three independent biological repeats. Data were analysed using Dunnett's multiple comparison test. *p < .05

To determine whether the expression of *VmGP* could be regulated by *Vm‐*milR37, the expression patterns of *Vm*‐milR37 and *VmGP* were analysed. According to the transcriptome data, *VmGP* was highly expressed during infection. To confirm this result, the expression of *VmGP* was analysed by quantitative reverse transcription PCR (RT‐qPCR). Consistent with transcriptome information, *VmGP* was up‐regulated during the infection process, thus differing from the expression profile of *Vm*‐milR37 (Figure 5a). Furthermore, the expression level of *VmGP* was significantly suppressed in *Vm*‐milR37 overexpression transformants both in vitro and in planta (Figure 5b,c). To further confirm the regulatory mechanism of *Vm*‐milR37, the overexpression construct of a mutated *Vm*‐milR37 (Mut‐R37), with mismatches to the sequence of *VmGP*, was generated (Figure 5d). The Mut‐R37 overexpression construct was transformed into wild‐type *V*. *mali* and three corresponding transformants (Mut‐R37‐1, Mut‐R37‐2, and Mut‐R37‐3) were obtained (Figure S7). The *VmGP* transcript level was quantified in Mut‐R37 overexpression transformants in vitro and during infection. Compared with the wild type, *VmGP* did not show a significant change in Mut‐R37 overexpression transformants in vitro and in planta (Figure 5e,f). These results indicate that *Vm*‐milR37 can suppress the expression of *VmGP* in a sequence‐specific manner.

**FIGURE 6:**
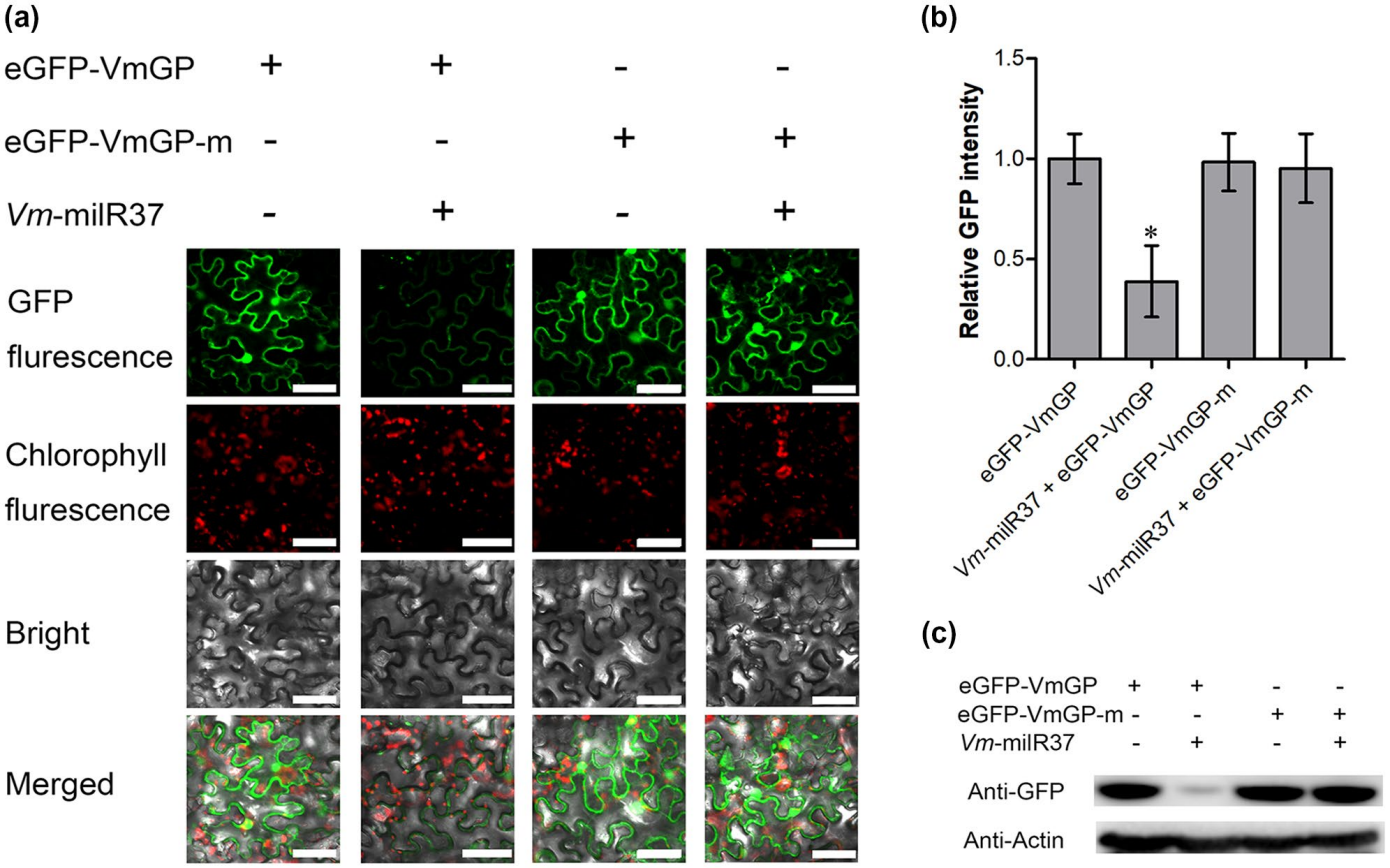
*Vm*‐milR37 suppresses the expression of *VmGP* in *Nicotiana benthamiana* by coinjection. (a) Coinfiltration of 35S:eGFP‐VmGP or 35S:eGFP‐VmGP mutated target site (35S:eGFP‐VmGP‐m) and 35S:*Vm*‐milR37 precursor in *N. benthamiana*. Confocal images were taken at 48 hr after *Agrobacterium* infiltration. (b) Green fluorescent protein (GFP) fluorescence intensity was quantified using confocal microscopy. Relative GFP intensity was normalized to the GFP intensity mean of eGFP‐VmGP. Error bars represent the *SD* of 30 *N. benthamiana* cells. Data were analysed using Dunnett's multiple comparison test. *p < .05. (c) Western blot analysis of eGFP‐VmGP and eGFP‐VmGP‐m. Identical amounts of proteins were loaded. Anti‐GFP and anti‐actin antibodies were used for western blot analysis. The amount of actin was used as the loading control. The coexpression experiment was independently repeated twice with similar results

Cotransformation assays in *Nicotiana benthamiana* leaves were applied to verify the accuracy of degradome sequencing using green fluorescent protein (*GFP*) as a reporter gene. The GFP fluorescence was observed in the leaves infiltrated with *Agrobacterium tumefaciens* GV3101 pCAMBIA1302‐eGFP‐VmGP and *A. tumefaciens* GV3101 pCAMBIA1302‐eGFP‐VmGP‐m (a construct containing a mutation in the target site of *VmGP*) (Figure S8). GFP fluorescence was also observed in leaves coinfiltrated with GV3101 pCAMBIA1302‐*Vm*‐milR37 and GV3101 pCAMBIA1302‐eGFP‐VmGP‐m. Almost no GFP fluorescence was observed in the leaves coinfiltrated with GV3101 pCAMBIA1302‐*Vm*‐milR37 and GV3101 pCAMBIA1302‐eGFP‐VmGP (Figure 6a). Fluorescence intensity, which indicates the expression of eGFP‐VmGP and eGFP‐VmGP‐m, was quantified by assessing 30 independent plant cells. The results showed that fluorescence intensity was much lower in the leaves coinfiltrated with GV3101 pCAMBIA1302‐*Vm*‐milR37 and GV3101 pCAMBIA1302‐eGFP‐VmGP. No significant difference was observed in other treatments (Figure 6b). To confirm the result of histological observation, the amount of GFP was assessed by western blotting in leaves infiltrated with different vectors. The amount of GFP in the leaves coinfiltrated with GV3101 pCAMBIA1302‐*Vm*‐milR37 and GV3101 pCAMBIA1302‐eGFP‐VmGP was much lower than in other treated leaves (Figure 6c). Thus, *Vm*‐milR37 decreased the expression of *VmGP* by cleaving the target fragment to a large extent.

### 2.5 *VmGP* contributes to the pathogenicity by affecting the oxidative stress response

**FIGURE 7:**
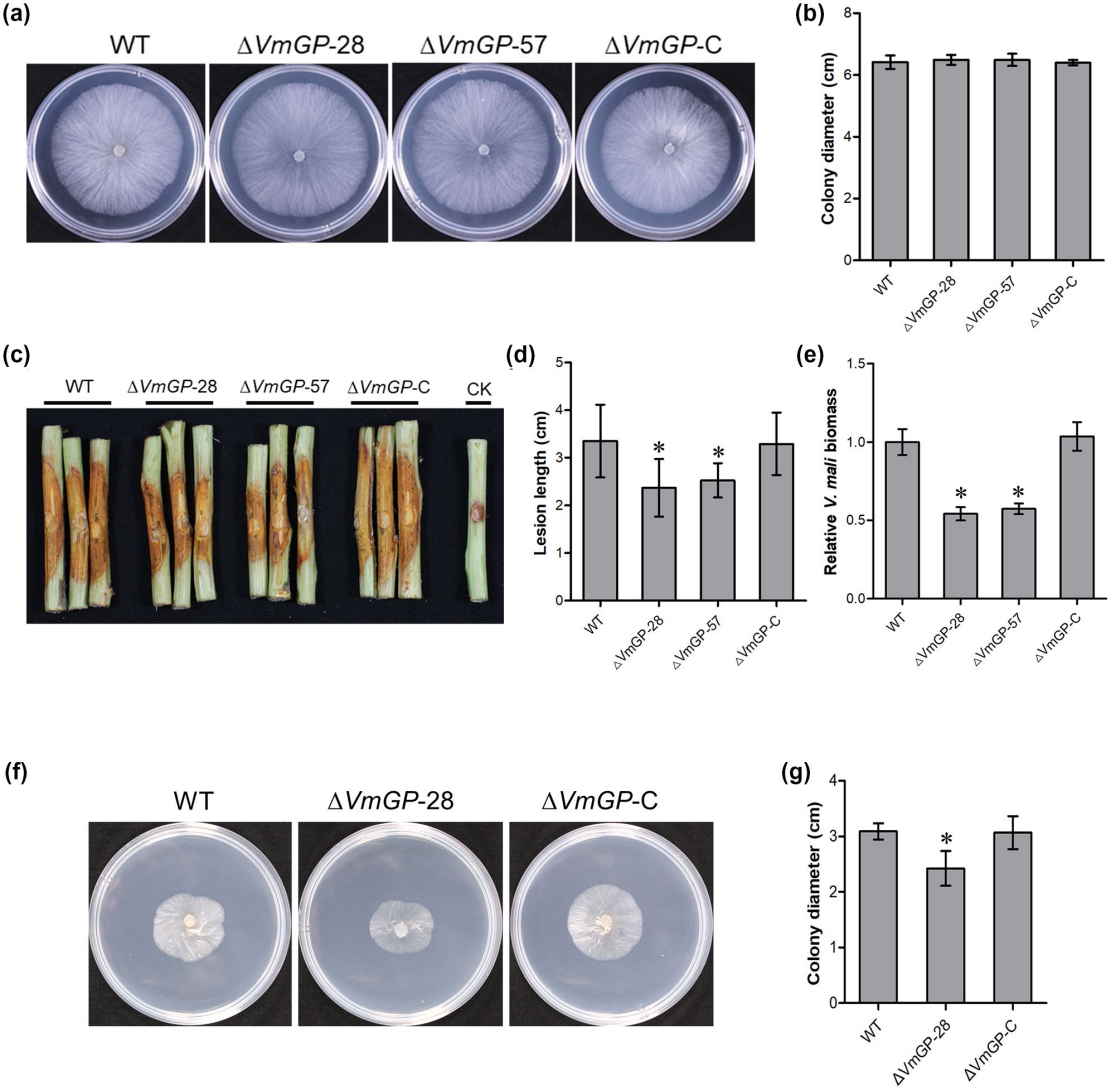
Deletion of *VmGP* reduces the pathogenicity of *Valsa mali*. (a) Colony morphology of wild type (WT), *VmGP* deletion mutants, and complementation strain after 48 hr incubation. (b) Colony diameters of WT, *VmGP* deletion mutants, and complementation strain aafter 48 hr incubation. Data represent mean ± *SD*. The experiment was repeated three times, each time with three plates. (c) and (d) Pathogenicity test of WT, *VmGP* deletion mutants, and complementation strain at 4 days postinoculation. Three representative diseased twigs are shown. The pathogenicity test was independently repeated three times, each time with four replicates. CK, negative control. Data represent mean ± *SD*. (e) *V. mali* biomass was measured with quantitative PCR using *V*. *mali*‐specific *VmG6PDH* primers. *V*. *mali* biomass was normalized to the mean of the WT. Data are mean ± *SD* of three technical replicates. Similar results were obtained from three biological repeats. (f) WT, *VmGP* deletion mutant, and complementation strain on potato dextrose agar (PDA) + 0.05% (vol/vol) H_2_O_2_. Photographs were taken after 48 hr incubation. (g) Colony diameters of WT, deletion mutant of *VmGP,* and complementation mutant on PDA + 0.05% (vol/vol) H_2_O_2_ after 48 hr incubation. Data represent mean ± *SD*. The experiment was repeated three times, each time with three plates. Significant difference was determined using Dunnett's multiple comparison test. *p < .05

To determine the function of *VmGP* in vegetative growth and pathogenicity, deletion mutants of *VmGP* were generated (Figure S9a). There was no distinct difference in vegetative growth between deletion mutants and the wild type (Figure 7a,b). Deletion of *VmGP* led to significantly reduced lesion sizes on twigs as compared with those caused by the wild type (Figure 7c,d). Reintroduction of *VmGP* to the *VmGP* deletion mutant restored the pathogenicity to the level of the wild type (Figures 7c,d and S9). *V*. *mali* biomass in twigs infected by *VmGP* deletion mutants was significantly reduced compared with those infected with the wild‐type and complementation transformant (Figure 7e). These results suggest that *VmGP* is required for the full pathogenicity of *V*. *mali*.

As *VmGP* contains typical glutathione peroxidase conserved domains, which are associated with reactive oxygen metabolism, oxidative stress analysis was investigated by measuring the growth rate of wild‐type and mutant strains on potato dextrose agar (PDA) supplemented with H_2_O_2_. The vegetative growth of *VmGP* deletion mutant was significantly inhibited. When *VmGP* was reintroduced into the deletion mutant, the defect in the oxidative stress response of *VmGP* deletion mutant was restored (Figure 7f,g).

**FIGURE 8:**
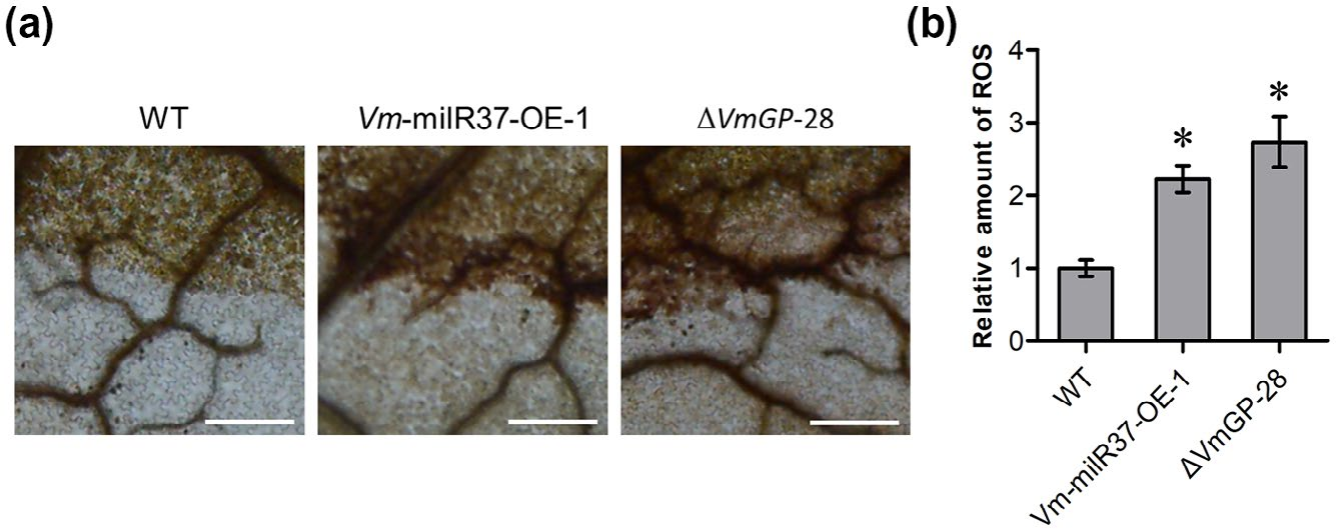
*Vm*‐milR37 and *VmGP* are involved in the oxidative stress response during *Valsa mali* infection. (a) Apple leaves inoculated with *Vm*‐milR37 overexpression (OE) transformant and *VmGP* deletion mutant exhibited enhanced reactive oxygen species (ROS) accumulation as compared with the wild type (WT). ROS in apple leaves were detected with 3,3′‐diaminobenzidine (DAB) staining at 24 hr postinoculation. (b) ImageJ software was used to quantify ROS accumulation at *V*. *mali* invasion sites. Mean ± *SD* was calculated from four biological repeats. Statistical significant difference was determined using Dunnett's multiple comparison test as compared to the WT. *p < .05

To determine whether *Vm*‐milR37 and *VmGP* are involved in the oxidative stress response during *V. mali* infection, the accumulation of reactive oxygen species (ROS) in apple leaves inoculated with wild type, *Vm*‐milR37 overexpression transformants, and the *VmGP* deletion mutant were measured. Compared with the control (wild type), apple leaves inoculated with *Vm*‐milR37 overexpression transformants and the *VmGP* deletion mutant displayed increased accumulation of ROS (Figure 8). Thus, we conclude that enhanced expression of *VmGP* regulated by *Vm*‐milR37 contributes to the pathogenicity by enhancing oxidative stress responsiveness during infection.

## 3 DISCUSSION

miRNAs were thought to be absent in fungi until a similar small RNA in *Neurospora* was identified to be milRNA (Lee et al., 2010). In contrast to research on plant and animal miRNAs, research on fungal milRNAs is less advanced. Many fungal milRNAs have been sequenced recently, such as from *Metarhizium anisopliae* (Zhou et al., 2012b), *Penicillium marneffei* (Lau et al., 2013), and *Aspergillus flavus* (Bai et al., 2015). milRNAs of some plant‐pathogenic fungi have been isolated, and these were predicted to be associated with vegetative growth and development, and pathogenicity by inhibiting the expression of endogenous genes, even using cross‐kingdom regulation (Chen et al., 2015; Jin et al., 2019; Lin et al., 2016; Liu et al., 2016; Wang et al., 2017; Zhou et al., 2012a). However, the detailed functions and regulatory mechanisms of milRNAs are still largely unknown. In this study, a milRNA, *Vm*‐milR37, was isolated from the plant‐pathogenic fungus *V. mali*. The function and regulatory mechanism of *Vm*‐milR37 were confirmed to be associated with pathogenicity by regulating the expression of *VmGP*.

The precursor of *Vm*‐milR37 was generated from an endogenous transcript that could fold to a typical hairpin structure, which meets the criterion for defining a fungal milRNA (Lee et al., 2010). *Vm*‐milR37 was specifically expressed in mycelium, and nearly no expression was detected during the host infection process. Thus, we speculated that *Vm*‐milR37 may play an important role in vegetative growth and pathogenicity. To analyse the function of miRNAs or milRNAs, overexpression is an important and widely used method (Jin et al., 2019; Li et al., 2013; Xu et al., 2020). Based on overexpression of *Vm*‐milR37 and pathogenicity assays, *Vm*‐milR37 was confirmed to be involved in the pathogenicity of *V*. *mali*.

milRNAs function by regulating the corresponding target genes (Bartel, 2004). Thus, target identification is critical for exploring the regulatory mechanism of milRNAs. In this study, *Vm*‐milR37 negatively regulated the pathogenicity of *V. mali*. As *Vm*‐milR37 did not show any expression during pathogen infection, it is not possible for *Vm*‐milR37 to suppress the plant immunity in a cross‐kingdom way like *Bc‐*siR3.1, *Bc‐*siR3.2, *Bc‐*siR5, *Bc‐*siR37 (Wang et al, 2017b; Weiberg et al., 2013), and *Pst*‐milR1 (Wang et al., 2017a). Thus, we speculated that *Vm*‐milR37 could contribute to pathogenicity by regulating an endogenous gene. A lack of effective methods to predict endogenous target genes of milRNAs limits the exploration of milRNA function in fungi (Torres‐Martínez & Ruiz‐Vázquez, 2017).

In plants and animals, miRNAs play important regulatory roles by targeting mRNAs for cleavage or translational repression (Bartel, 2004). In fungi, the regulatory mechanism of milRNAs is largely unknown. In *V. dahliae,* milRNA1 is involved in fungal virulence by transcriptional repression (Jin et al., 2019). *Arabidopsis* sRNAs and cotton miRNAs can be transported into fungal cells and silence fungal target transcripts by mRNA cleavage, which indicates that sRNA‐mediated mRNA cleavage exists in fungi (Cai et al., 2018; Zhang et al., 2016). In this study, *VmGP* was identified to be the target gene of *Vm*‐miR37, with subsequent mRNA cleavage. As core components of the RISC, AGOs can perform a cleavage function when sRNAs guide them by binding with the corresponding target genes (Azlan et al., 2016). Based on the published genome sequence of *V. mali*, three proteins have been idenitfied as VmAGOs (Yin et al., 2015). If milRNAs play roles in the mRNA cleavage pathway, the corresponding mRNA ends will have a 5′ phosphate and this character can be used for identification of milRNA target genes (German et al., 2008). The target genes of milRNAs in *V. mali* were detected based on degradome sequencing, and they could be regulated by milRNAs by mRNA cleavage (Xu et al., 2020). Degradome sequencing of *F. oxysporum* and *F. graminearum* also revealed that RNAi‐mediated gene suppression can function at the posttranscriptional level (Chen et al., 2014; Son et al., 2017). Thus, mRNA cleavage mediated by endogenous milRNA may be a critical regulatory mechanism in fungi.

*VmGP* encodes a glutathione peroxidase, and it was demonstrated to play a critical role in pathogenicity and the oxidative stress response of *V. mali*. In *Magnaporthe oryzae*, a GPx has been shown to be required for H_2_O_2_ resistance and fungal virulence (Huang et al., 2011). The glutathione peroxidase of *Alternaria alternata* is associated with ROS resistance and full virulence (Yang et al., 2016). Plant cells trigger an oxidative burst with a rapid increase of ROS production to defend against pathogen infection (Auh & Murphy, 1995). To infect successfully, pathogens have to increase their tolerance to these ROS. Glutathione peroxidase is a key enzyme to degrade H_2_O_2_ (Aung‐Htut et al., 2011). Previous studies have demonstrated that the ability to detoxify ROS is required for *A. alternata* survival and pathogenesis (Chung, 2012; Lin et al., 2009). Thus, we speculated that *VmGP* may contribute to full pathogenicity by enhancing tolerance to H_2_O_2_ from the host plant.

It is well known that gene expression can be regulated at the transcriptional level by transcription factors and by epigenetic regulation in pathogenic fungi to adapt to diverse environments (van der Does & Rep, 2017; Soyer et al., 2014; Tan & Oliver, 2017). Gene expression regulation at the posttranscriptional level has been found to exist in most eukaryotes (Ghildiyal & Zamore, 2009; Katiyar‐Agarwal & Jin, 2010). In pathogenic fungi, many virulence genes have been predicted and confirmed to be regulated by sRNAs (Gowda et al., 2010; Guo et al., 2019; Jin et al., 2019; Raman et al., 2017; Xu et al., 2020). We also found that many virulence genes of *V*. *mali* could be regulated by milRNAs (Xu et al., 2020). In this study, *VmGP*, as an important virulence gene, was further demonstrated to be regulated by *Vm*‐milR37 at the posttranscriptional level. When the fungus does not need to express the virulence gene, the fine‐tuning mode of sRNA is activated. We speculate that this mechanism is beneficial for the fungus to save energy to enhance the adaption capacity and pathogenicity.

Overall, this study demonstrates that a milRNA, *Vm*‐milR37 from *V. mali*, plays a critical role in pathogenicity by regulating the endogenous target gene *VmGP*, which contributes to the oxidative stress response during *V. mali* infection. These results provide important evidence to define the roles of milRNAs and their corresponding target genes in fungal pathogenicity.

## 4 EXPERIMENTAL PROCEDURES

### 4.1 Strains and growth conditions

The wild‐type strain of *V. mali* 03‐8 was used to generate transformants of *Vm*‐milR37 and *VmGP*. All the strains were cultured on PDA at 25 °C in the dark. *Escherichia coli* DH5α was cultured in lysogeny broth at 37 °C. *Agrobacterium tumefaciens* GV3101 was cultured in lysogeny broth at 28 °C.

### 4.2 Expression profiles of *Vm‐*milR37 and *VmGP*

Mycelial plugs (5 mm diameter) of *V. mali* were inoculated onto twigs of *Malus* × *domestica* 'Fuj' as described by Wei et al. (2010). To investigate the function of *Vm*‐milR37 and *VmGP* during the *V. mali–*apple bark interaction, the junction of healthy and infected apple bark tissue inoculated with *V. mali* for 6, 12, 24, 48, and 72 hr was collected. Samples of *V. mali* mycelium cultured for 3 days were collected as a control (0 hr postinoculation [hpi]) from PDA plates covered with a layer of cellophane.

Total RNA of each sample was extracted with TRIzol reagent (Invitrogen) following the manufacturer's instructions. RNA purity, concentration, and integrity were checked. First‐strand cDNA was synthesized using a reverse transcription (RT)‐PCR system (Promega) following the manufacturer's instructions. The expression level of *Vm*‐milR37 was detected by stem‐loop RT‐PCR described by Feng et al. (2012). Small nuclear RNA U6 of *V. mali* (*Vm*U6) was used as an internal control. The expression level of *VmGP* was measured followed the method described by Yin et al. (2013). *G6PDH* of *V. mali* was selected as the internal control. There were three biological replicates for each treatment. Primers used for RT‐qPCR are given in Table S1.

### 4.3 Generation of *Vm*‐milR37 and Mut‐R37 overexpression transformants

The precursor of *Vm*‐milR37 was amplified from *V. mali* genomic DNA using Phusion high‐fidelity DNA polymerase (New England Biolabs) and cloned into plasmid pDL2 using the ClonExpress‐II One Step Cloning Kit (Vazyme Biotech). The Mut‐R37 overexpression construct was generated using the Fast Site‐Directed Mutagenesis Kit (Tiangen) following the manufacturer's instructions and the *Vm*‐milR37 overexpression construct as the amplification template. Constructs were verified by sequencing and transformed into *V. mali* wild‐type strain 03‐8 as described above. Transformants were screened by PCR with primer pairs outside the cloning sites of pDL2. Relative expression profiles of *Vm*‐milR37 and *VmGP* were measured as described above. All primers used for gene deletion are given in Table S1.

### 4.4 Target identification of *Vm*‐milR37

Based on the degradome sequencing results, the 3′ untranslated region (UTR) region of VM1G_06866 was identified as the target of *Vm*‐milR37. To verify whether the expression of VM1G_06866 could be suppressed by *Vm*‐milR37, the precursor of *Vm*‐milR37 and the 3′ UTR of VM1G_06866 were cloned into pCAMBIA1302 with *GFP* as a reporter gene, and the recombinant vectors were cotransformed into the same site of *N. benthamiana* leaves using the *Agrobacterium*‐mediated transfection system described by Weiberg et al. (2013). Confocal images were taken at 48 hr post‐*Agrobacterium* infiltration. The GFP fluorescence intensity quantified by confocal microscopy represented the expression of the target gene. Thirty independent *N. benthamiana* cells were used to detect the fluorescence intensity. Data were analysed using Dunnett's multiple comparison test (*p* < .05). To further verify the expression of GFP, anti‐GFP and anti‐actin antibodies (Sungene Biotech) were used for western blot analysis. Horseradish peroxidase‐conjugated goat antimouse IgG (Cwbiotech) was used as a secondary antibody. The coexpression experiment was repeated twice independently. All primers used for coexpression are given in Table S1.

### 4.5 Sequence alignment and phylogenetic analysis

The full length of the target gene was isolated based on the results of degradome sequencing and genome information. The corresponding coding sequence was deduced from the coding sequence, and the closest homologous sequences in fungi and plants were identified by BlastP (http://www.ncbi.nlm.nih.gov/BLAST/). The conserved domain was predicted by NCBI’s conserved domain database (http://www.ncbi.nlm.nih.gov/Structure/cdd/wrpsb.cgi). Multiple alignment of protein sequences was made with the program CLUSTAL X2. The phylogenetic comparison of homologous sequences from GenBank (http://www.ncbi.nlm.nih.gov/) was constructed with the neighbour‐joining method using MEGA 7. The bootstrap value was set as 1,000.

### 4.6 Generation of target gene deletion mutants and complementation transformants

The *NEO* gene was a selected as marker gene to perform target gene deletion. The *NEO* gene fragment was amplified from plasmid pFL2 with primers Neo‐F and Neo‐R. The *NEO* fragment was fused with upstream and downstream flanking sequences of the target gene by double‐joint PCR (Yu et al., 2004). The gene‐replacement construct was transformed into protoplasts of *V. mali* as previously described (Gao et al., 2011). Each putative single gene deletion mutant was verified by PCR with four primer pairs to detect the target gene, the *NEO* gene, upstream‐*NEO* fusion segment, and *NEO*‐downstream fusion segment.

To generate the complementation transformants of target gene deletion mutants, the full‐length target gene with upstream 2,000 bp was amplified from genomic DNA and cloned into plasmid pDL2 using the yeast gap repair approach (Bruno et al., 2004). The recombinant construct was then transformed into protoplasts of the gene deletion mutant. Complemented transformants were selected using geneticin (G418) and hygromycin, and confirmed by PCR. All primers used for gene deletion are given in Table S1.

### 4.7 Vegetative growth, pathogenicity, and fungal biomass assays

The vegetative growth of gene deletion mutants and overexpression transformants was assayed as previously described (Xu et al., 2018). The tests were performed three times and each experiment included three replicates. Pathogenicity assays were performed on Fuji apple twigs as described (Wei et al., 2010). Lesion length was measured at 4 days postinoculation. The pathogenicity test was repeated three times and each experiment included four replicates. For *V*. *mali* biomass assays, samples of 0.4 g apple twig tissues, including the infected tissues and healthy tissue, were collected. Genomic DNA was isolated with the Super Plant Genomic DNA kit (Polysaccharides and Polyphenolics‐rich; Tiangen). *V. mali* biomass was measured with quantitative PCR using *V*. *mali*‐specific *VmG6PDH* primers. The biomass assay was independently performed three times, each time with three technical replicates.

### 4.8 Oxidative stress test

Mycelial plugs (5 mm diameter) from the edge of growing colonies of *V. mali* strain 03‐8 and gene deletion mutants were inoculated on PDA supplemented with 0.05% H_2_O_2_. The colony diameter was determined after 2 days’ incubation. The test was performed three times and each experiment included three replicates.

### 4.9 ROS staining in apple leaves

*V. mali* strains were inoculated on apple leaves as previously described (Wei et al., 2010). At 24 hpi, apple leaves around the inoculation points were cut into 1 cm^2^ pieces and immediately immersed in 1 mg/ml 3,3′‐diaminobenzidine (DAB, pH 3.8). After staining for 8 hr in the light, apple pieces were decoloured using 3:1 (vol/vol) ethanol:chloroform containing 0.15% trichloroacetic acid and saturated chloral hydrate solution. Photographs were taken using a DP72 camera (Olympus). ROS accumulation in *V*. *mali* invasion sites was quantified with ImageJ software. The relative amount of ROS was normalized to the mean of leaves inoculated with the wild type. The test was performed three times.

## AUTHOR CONTRIBUTIONS

H.F., M.X., and L.H. designed the research. M.X. and Y.G. mainly contributed to the all experiments. J.L., F.G., and Y.G. assisted with specific experiments. H.F. prepared the manuscript and L.H. revised the manuscript. None of the authors have conflicts of interest with this manuscript.

## Supporting information


**FIGURE S1** The secondary structure of *Vm*‐milR37 forms a hairpin structure. The sequence underlined in red indicates the mature sequence of *Vm*‐milR37

**FIGURE S2** Detection of *Vm*‐milR37 overexpression transformants by PCR

**FIGURE S3** Conserved domain of VmGP

**FIGURE S4** VmGP is a unique glutathione peroxidase in *Valsa* *mali* by BlastP analysis

**FIGURE S5** No signal peptide is found in VmGP using SignalP v. 5.0 prediction

**FIGURE S6** VmGP is likely to be located in the mitochondrion using WoLF PSORT prediction

**FIGURE S7** Detection of Mut‐R37 overexpression transformants (Mut‐R37‐1, Mut‐R37‐2, and Mut‐R37‐3) using primer pair pDL2‐mexp‐JC‐F and Mut‐R37‐OE‐R. The genomic DNA wild type and transformant with the empty vector (EV) was used as control

**FIGURE S8** Sequence alignment of *Vm*‐milR37 with *VmGP* target region (*VmGP*) and mutated *VmGP* target region (*VmGP*‐m)

**FIGURE S9** Detection of *VmGP* deletion mutants and complementation strain. (a) Detection of *VmGP* deletion mutants by four steps of PCR. Lane 1, PCR product amplified with primer pair VmGP‐5F/6R designed from inner of *VmGP* was used to ascertain the deletion of target gene. Genomic DNA of the wild type (WT) was used as positive control. Sterile double‐distilled water was used as negative control. Lane 2, PCR product amplified with primer pair G852‐F/G850‐R designed from inner of *NEO* was used to ascertain the insertion of *NEO*. Lane 3, PCR product amplified with primer pair VmGP‐7F/G855‐R was used to ascertain the targeted homologous recombination upstream of *VmGP*. Lane 4, PCR product amplified with primer pair G856‐F/VmGP‐8R was used to ascertain the targeted homologous recombination downstream of *VmGP*. (b) Detection of *VmGP* deletion mutants and complementation strain by reverse transcription‐PCR. The *Valsa mali* housekeeping gene *VmG6PDH* was used as control

**TABLE S1** Primers used in this study

## Data Availability

The data that support the findings of this study are available from the corresponding author upon reasonable request.
