# Supplementary figures and images for: Vm‐milR37 contributes to pathogenicity by regulating glutathione peroxidase gene VmGP in Valsa mali
Source: Mol Plant Pathol. 2020 Dec 5;22(2):243–54. doi: 10.1111/mpp.13023 (PMC7814965; doi:10.1111/mpp.13023)

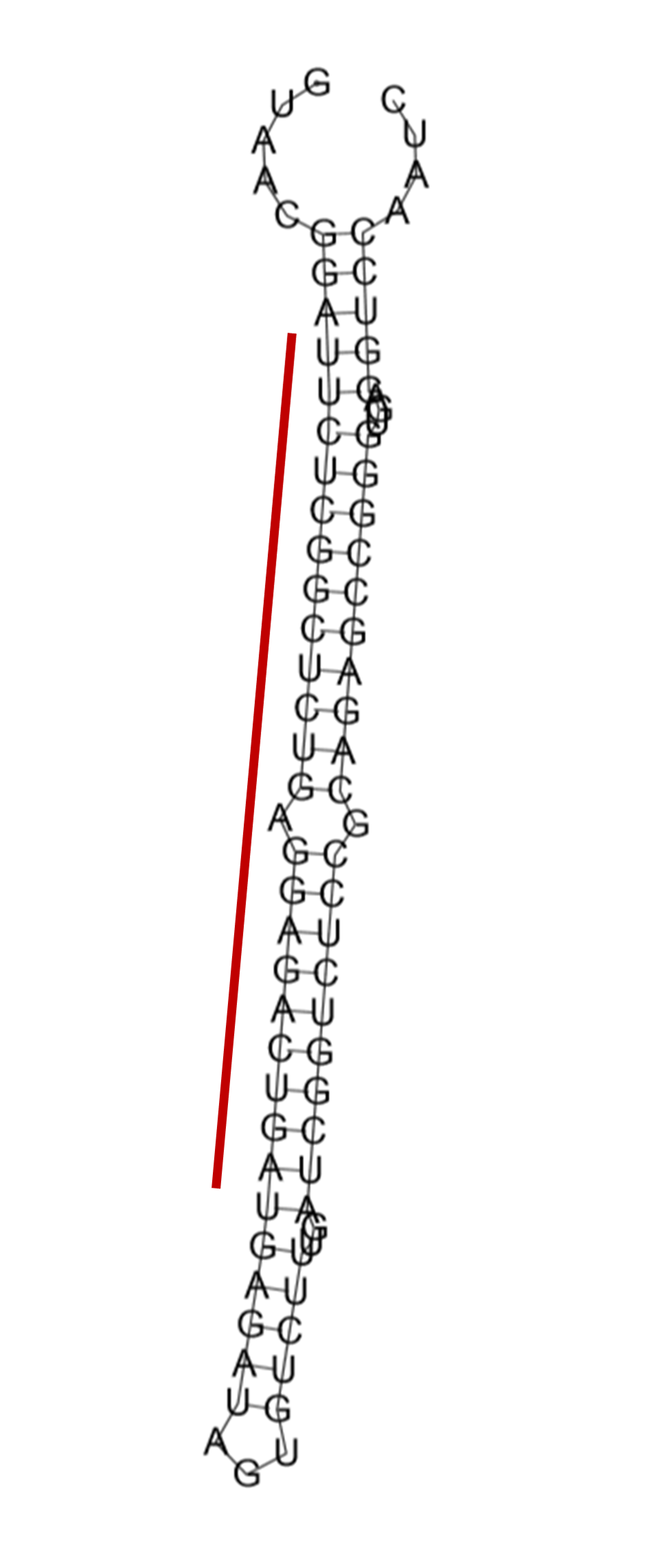

Supplement: Supplementary file 1 — FIGURE S1 The secondary structure of Vm‐milR37 forms a hairpin structure. The sequence underlined in red indicates the mature sequence of Vm‐milR37 [file MPP-22-243-s001.TIF]

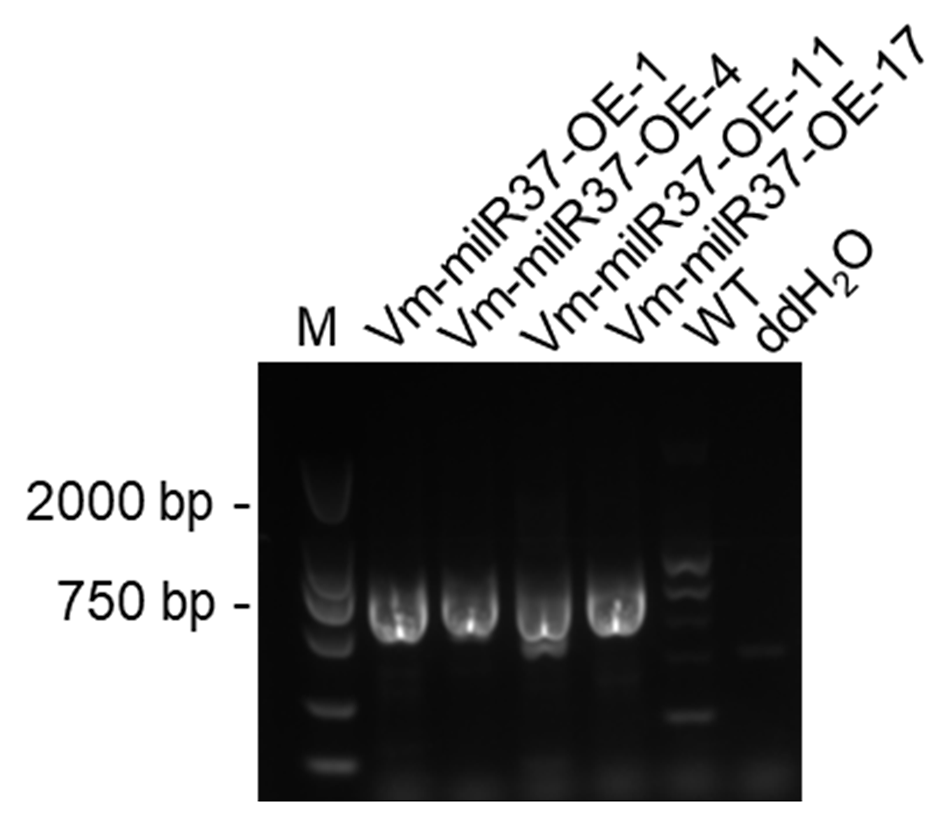

Supplement: Supplementary file 2 — FIGURE S2 Detection of Vm‐milR37 overexpression transformants by PCR [file MPP-22-243-s002.TIF]

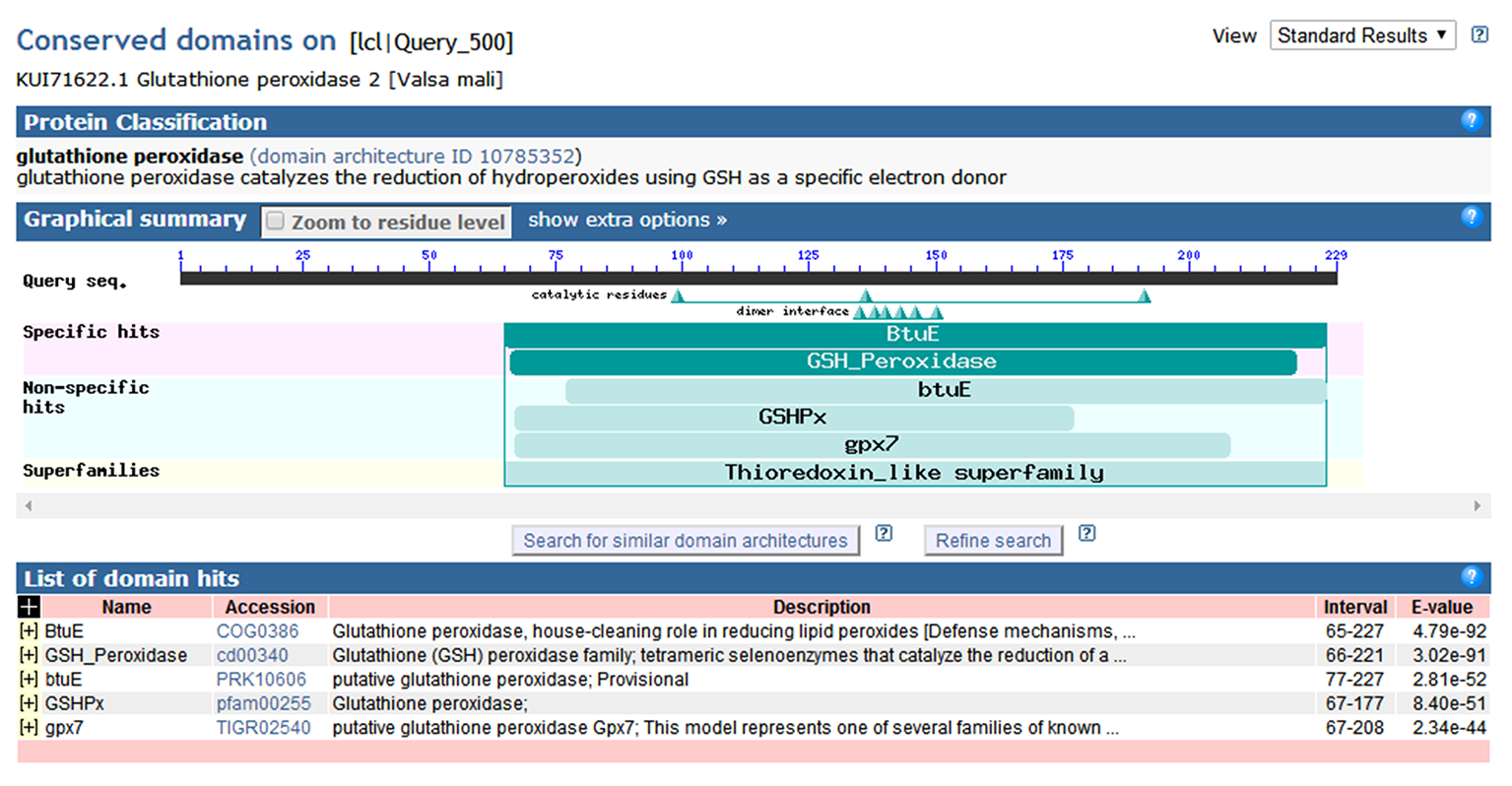

Supplement: Supplementary file 3 — FIGURE S3 Conserved domain of VmGP [file MPP-22-243-s003.TIF]

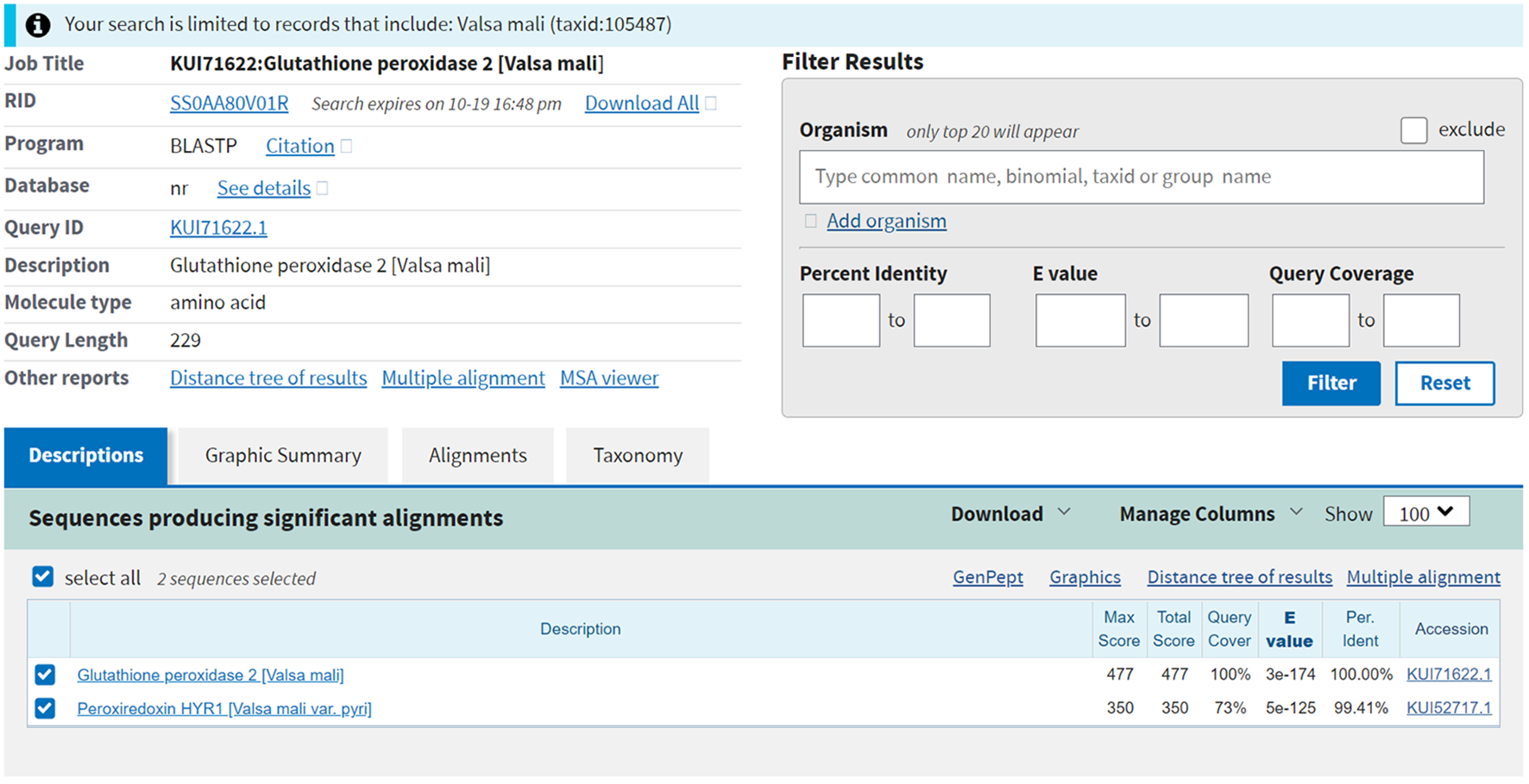

Supplement: Supplementary file 4 — FIGURE S4 VmGP is a unique glutathione peroxidase in Valsa mali by BlastP analysis [file MPP-22-243-s004.TIF]

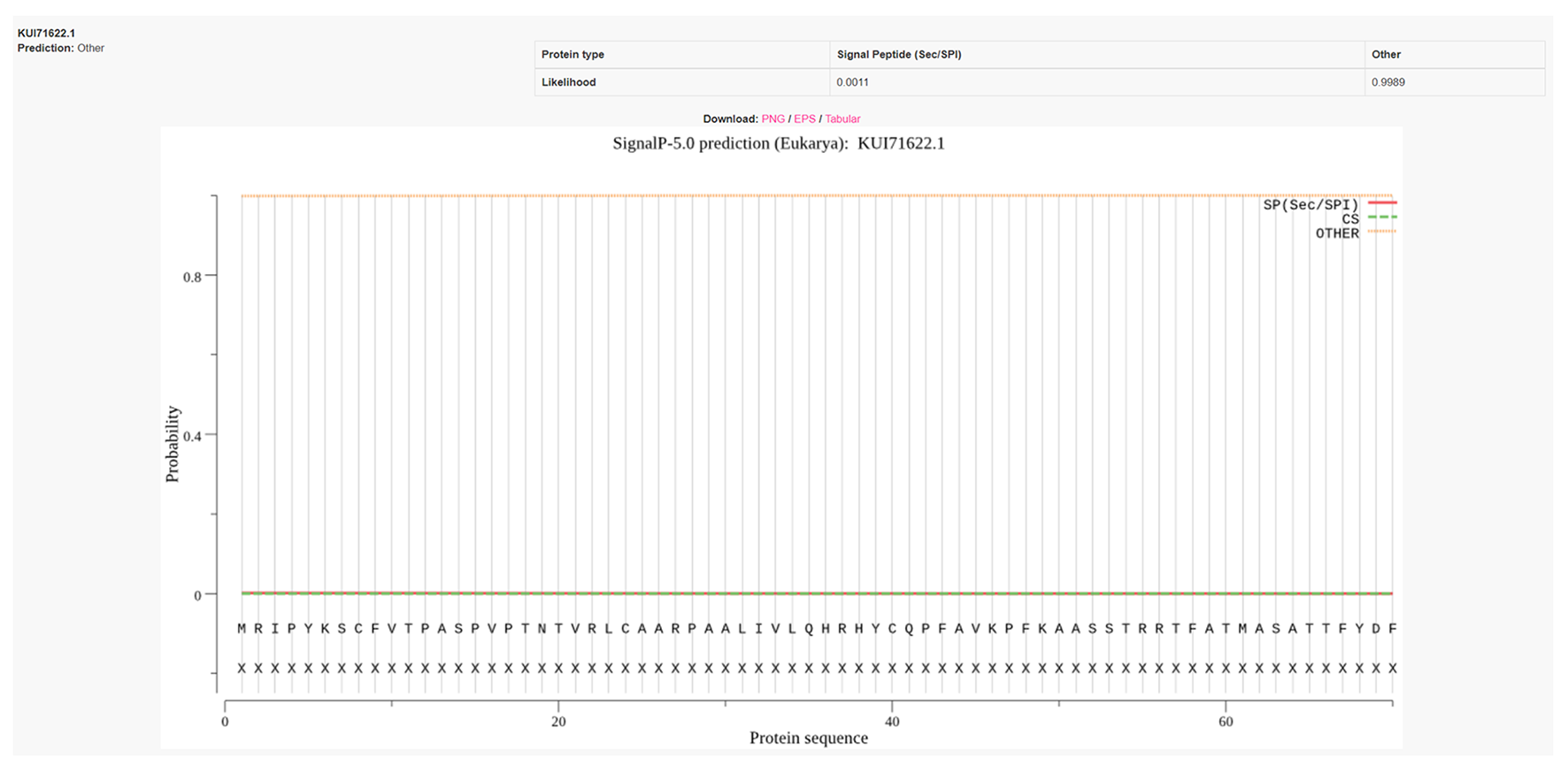

Supplement: Supplementary file 5 — FIGURE S5 No signal peptide is found in VmGP using SignalP v. 5.0 prediction [file MPP-22-243-s005.TIF]

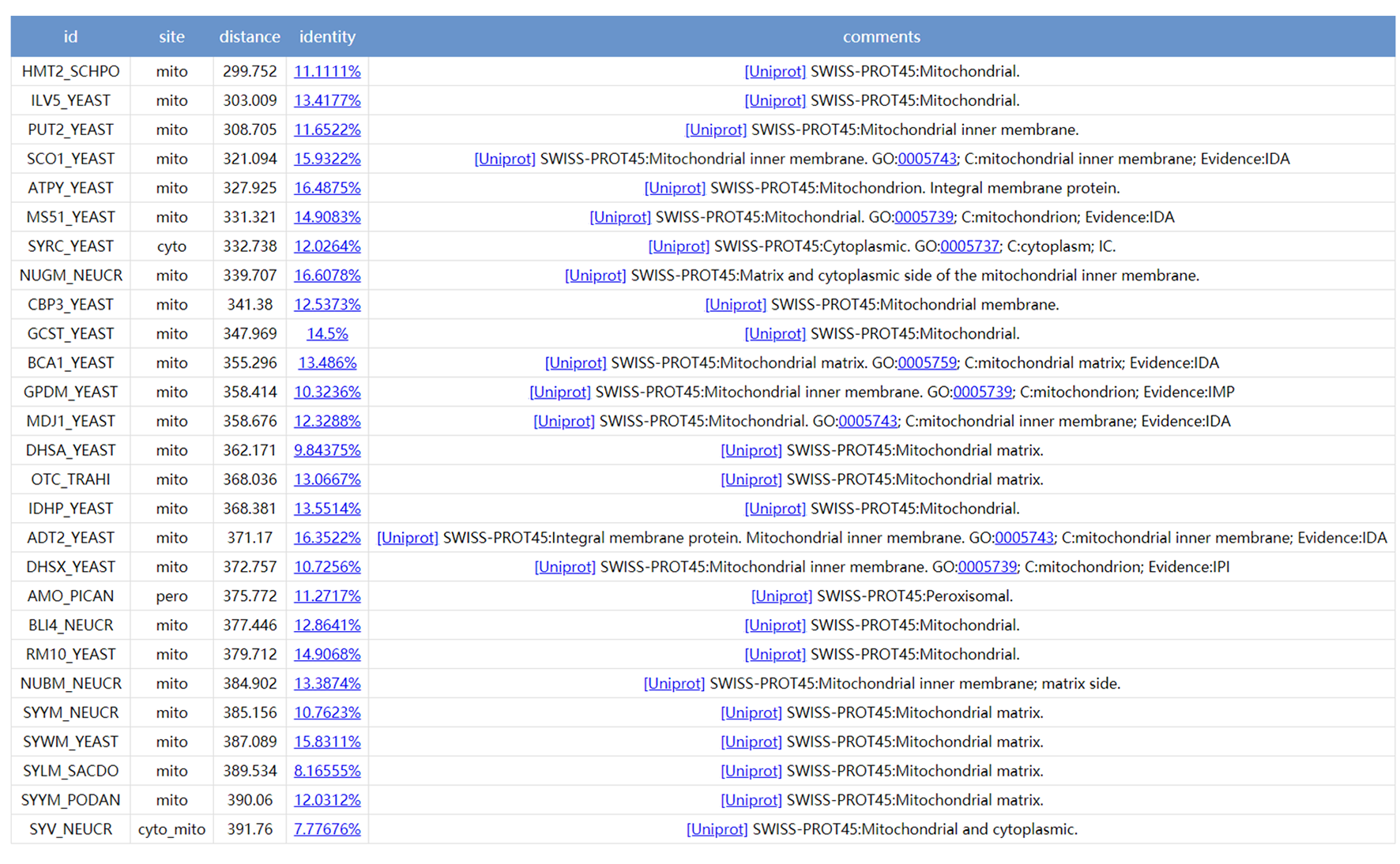

Supplement: Supplementary file 6 — FIGURE S6 VmGP is likely to be located in the mitochondrion using WoLF PSORT prediction [file MPP-22-243-s006.TIF]

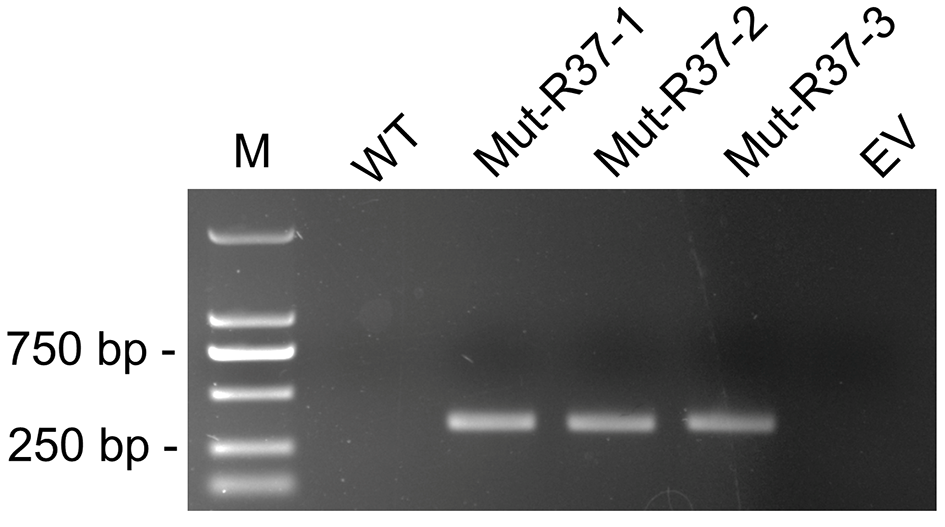

Supplement: Supplementary file 7 — FIGURE S7 Detection of Mut‐R37 overexpression transformants (Mut‐R37‐1, Mut‐R37‐2, and Mut‐R37‐3) using primer pair pDL2‐mexp‐JC‐F and Mut‐R37‐OE‐R. The genomic DNA wild type and transformant with the empty vector (EV) was used as control [file MPP-22-243-s007.TIF]

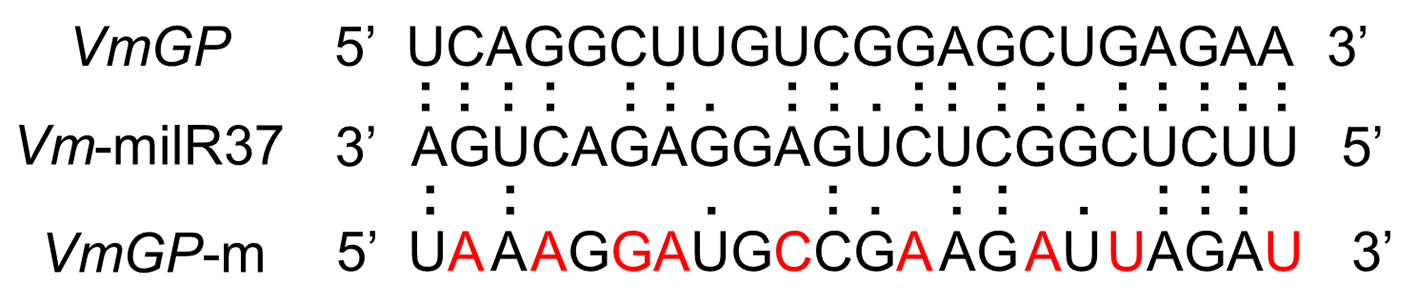

Supplement: Supplementary file 8 — FIGURE S8 Sequence alignment of Vm‐milR37 with VmGP target region (VmGP) and mutated VmGP target region (VmGP‐m) [file MPP-22-243-s008.TIF]

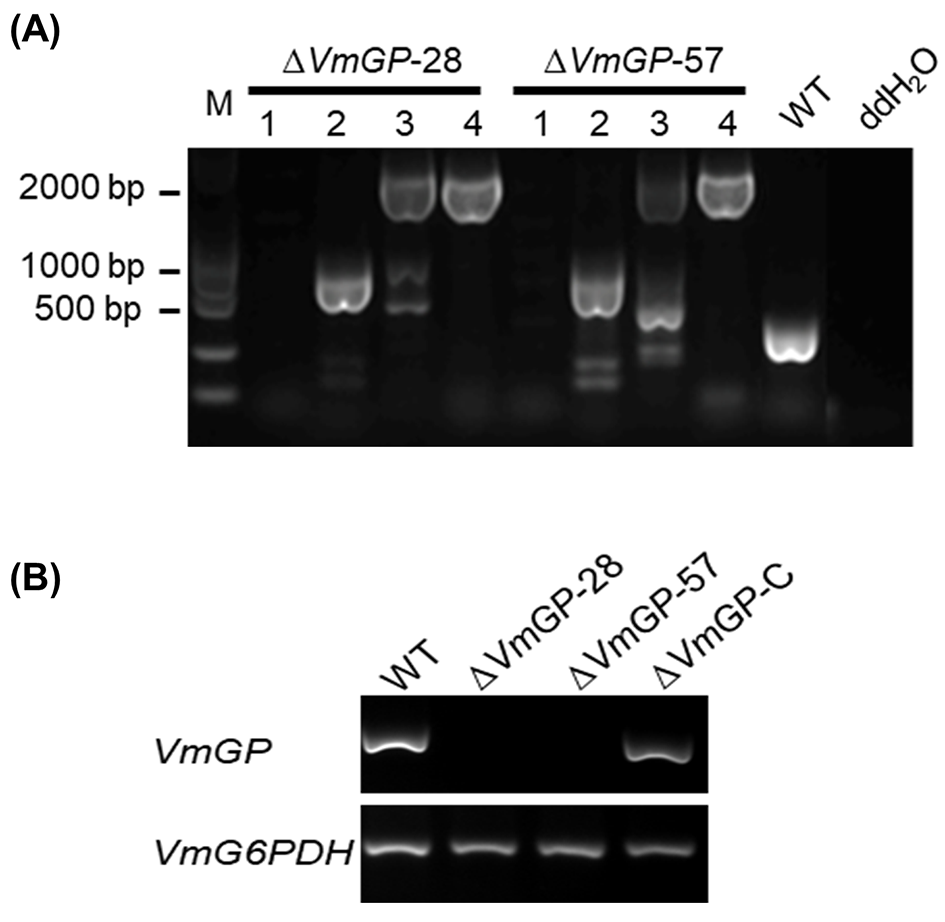

Supplement: Supplementary file 9 — FIGURE S9 Detection of VmGP deletion mutants and complementation strain. (a) Detection of VmGP deletion mutants by four steps of PCR. Lane 1, PCR product amplified with primer pair VmGP‐5F/6R designed from inner of VmGP was used to ascertain the deletion of target gene. Genomic DNA of the wild type (WT) was used as positive control. Sterile double‐distilled water was used as negative control. Lane 2, PCR product amplified with primer pair G852‐F/G850‐R designed from inner of NEO was used to ascertain the insertion of NEO. Lane 3, PCR product amplified with primer pair VmGP‐7F/G855‐R was used to ascertain the targeted homologous recombination upstream of VmGP. Lane 4, PCR product amplified with primer pair G856‐F/VmGP‐8R was used to ascertain the targeted homologous recombination downstream of VmGP. (b) Detection of VmGP deletion mutants and complementation strain by reverse transcription‐PCR. The Valsa mali housekeeping gene VmG6PDH was used as control [file MPP-22-243-s009.TIF]
